# Real-World Comparative Study of Atezolizumab-Based Chemotherapy Regimens in Advanced Non-Small Cell Lung Cancer

**DOI:** 10.3390/cancers17223630

**Published:** 2025-11-12

**Authors:** Ayaka Ohiwa, Tadashi Nishimura, Tadashi Sakaguchi, Hajime Fujimoto, Shuji Kodama, Atsushi Fujiwara, Hiroki Nakahara, Taichi Isobe, Takaya Hirai, Akihiko Yagi, Aiko Ebihara, Hidenori Ibata, Osamu Hataji, Masamichi Yoshida, Hisamichi Yuda, Taro Yasuma, Corina N. D’Alessandro-Gabazza, Esteban C. Gabazza, Tetsu Kobayashi

**Affiliations:** 1 Department of Pulmonary Medicine, Kuwana City Medical Center, Kotobukicho 3-11, Kuwana 511-0061, Mie, Japan; 2 Department of Pulmonary Medicine, Mie Chuo Medical Center, Hisaimyojin-cho 2158-5, Tsu 514-1101, Mie, Japan; 3 Respiratory Center, Matsusaka Municipal Hospital, Tonomachi 1550, Matsusaka 515-8544, Mie, Japan; 4 Department of Pulmonary and Critical Care Medicine, Mie University Faculty and Graduate School of Medicine, Edobashi 2-174, Tsu 514-8507, Mie, Japan; 5 Department of Pulmonary Medicine, Mie Prefectural General Medical Center, Hinaga 5450-132, Yokkaichi 510-8561, Mie, Japan; 6 Department of Pulmonary Medicine, Suzuka General Hospital, Yasuzuka-cho Yamanohana 1275-5, Suzuka 513-8630, Mie, Japan; 7 Department of Immunology, Mie University Faculty and Graduate School of Medicine, Edobashi 2-174, Tsu 514-8507, Mie, Japan

**Keywords:** immune checkpoint inhibitors, atezolizumab, bevacizumab, non-small cell lung cancer, chemotherapy, real-world clinical practice

## Abstract

Background/objective: Carboplatin, pemetrexed, and pembrolizumab are established as a key first-line regimen for metastatic non-small cell lung cancer, although selecting the optimal therapy for each patient remains challenging in real-world clinical practice. This retrospective multicenter study compared the efficacy and safety of two atezolizumab-based combination regimens, ACnP (carboplatin, nab-paclitaxel, atezolizumab) and ABCP (carboplatin, paclitaxel, bevacizumab, atezolizumab), in patients with non-small cell lung cancer in real-world clinical practice. Methods: A total of 91 patients treated between May 2018 and December 2023 at six Japanese hospitals were analyzed: 40 received ACnP and 51 received ABCP. Patient characteristics, treatment outcomes, and adverse events were compared, with subgroup analyses adjusted by inverse probability of treatment weighting using propensity scores. Results: The objective response rates were 55.0% with ACnP and 45.1% with ABCP. Median progression-free survival was 5.5 months for ACnP and 6.9 months for ABCP, while median overall survival was 16.2 and 18.3 months, respectively. Subgroup analyses showed significantly improved progression-free survival with ABCP in patients with brain metastases, liver metastases, EGFR-positive tumors, PD-L1-positive tumors, and impaired renal function (CCr < 45 mL/min). ABCP also conferred overall survival benefits in patients with brain and liver metastases. However, ACnP was associated with a lower incidence of neutropenia, peripheral neuropathy, and skin rash. Conclusions: These findings suggest that ABCP may offer superior efficacy in specific non-small cell lung cancer subgroups, while ACnP remains a valuable option for patients requiring a more tolerable safety profile.

## Simple Summary

Selecting the most appropriate first-line treatment for patients with advanced non-small cell lung cancer remains difficult in daily clinical practice, despite the availability of several effective combination therapies. This study compared two atezolizumab-based treatment regimens: one that included carboplatin, nab-paclitaxel, and atezolizumab, and the other that combined carboplatin, paclitaxel, bevacizumab, and atezolizumab, using real-world data from six hospitals in Japan. The aim was to clarify which regimen provides better outcomes and tolerability for different patient groups. The results suggest that the regimen containing bevacizumab may offer improved survival in patients with brain or liver metastases and in those with specific tumor profiles, while the nab-paclitaxel regimen caused fewer side effects such as nerve damage and low white blood cell counts. These findings may help physicians select more personalized and effective treatments for patients with advanced lung cancer in clinical practice.

## 1. Introduction

Lung cancer, comprising both small cell lung cancer (SCLC) and non-small cell lung cancer (NSCLC), remains one of the leading causes of cancer-related mortality worldwide [1]. Recent advancements in first-line treatment have been driven by the integration of immune checkpoint inhibitors (ICIs) into chemotherapy regimens, resulting in significantly improved patient outcomes [2,3,4,5]. Among these, the combination of carboplatin, pemetrexed, and pembrolizumab has amassed substantial evidence and is now widely recognized as a cornerstone in the front-line treatment of metastatic NSCLC [5,6,7]. Nevertheless, despite the increasing use of combination therapies, selecting the most appropriate regimen for individual patients remains a challenge in real-world clinical practice.

Within the established treatment landscape for advanced NSCLC, the IMpower150 regimen (atezolizumab, bevacizumab, carboplatin, and paclitaxel; ABCP) and the IMpower130 regimen (atezolizumab, carboplatin, and nab-paclitaxel; ACnP) have demonstrated considerable efficacy, particularly in defined patient subgroups [8,9]. These regimens provide viable alternatives for patients with contraindications to pemetrexed-based chemotherapy, such as those with renal dysfunction or resistance to epidermal growth factor receptor tyrosine kinase inhibitors (EGFR-TKIs) [10,11]. However, despite their shared clinical utility, direct real-world comparative analyses of their efficacy and safety profiles are lacking.

To address this gap and support more informed decision-making, this retrospective study systematically evaluates and compares clinical outcomes associated with the ACnP and ABCP regimens in routine practice. By identifying patient characteristics that may favor one regimen over the other, our findings aim to contribute to the development of more personalized treatment strategies for advanced NSCLC.

## 2. Materials and Methods

### 2.1. Study Population

Patients with a diagnosis of non-squamous and non-small cell lung cancer (NSCLC) who received either the ACnP or ABCP treatment regimens between May 2018 and December 2023 were enrolled from six medical centers across Japan. Patients with mutations in the epidermal growth factor receptor (EGFR), anaplastic lymphoma kinase (ALK), or other known driver genes were included. The chemotherapy protocol included carboplatin (area under the curve: 5 mg/mL·min), paclitaxel at a dose of 200 mg/m^2^ administered for up to four cycles, and bevacizumab at 15 mg/kg of body weight. Nab-paclitaxel was administered weekly at a dose of 100 mg/m^2^. The immune checkpoint inhibitor, atezolizumab, was administered at a fixed dose of 1200 mg every three weeks.

Patient data were extracted from electronic medical records. Key clinical outcomes, including overall response rate, disease control rate, progression-free survival (PFS), and overall survival (OS), were assessed and compared between the ACnP and ABCP cohorts. The incidence of treatment-related adverse events was also evaluated across both groups.

### 2.2. Statistical Analysis

Given the heterogeneity in baseline characteristics between the two treatment cohorts, direct comparisons of response rates, Kaplan–Meier survival distributions, and adverse event frequencies were not performed. Instead, these data are presented separately for each group. Subgroup analyses were subsequently conducted to delineate the patient populations in which each regimen demonstrated favorable therapeutic effects.

Tumor response was assessed in accordance with the Response Evaluation Criteria in Solid Tumors (RECIST), version 1.1, to calculate the overall response rate (ORR) and disease control rate (DCR). Progression-free survival (PFS) and overall survival (OS) were estimated using the Kaplan–Meier method, and categorical variables were compared using Fisher’s exact test.

To account for differences in baseline characteristics among subjects, background factor adjustment was performed using inverse probability of treatment weighting (IPTW), followed by subgroup analyses. Because inverse probability of treatment weighting (IPTW) is primarily used to estimate hazard ratios (HRs), it was applied exclusively for HR adjustment within the subgroup analyses. Subgroup analyses were performed using Cox proportional hazards regression models.

Hazard ratios (HRs) were estimated after adjustment for potential confounding variables, including age, sex, Eastern Cooperative Oncology Group performance status (ECOG PS), smoking history, histological subtype, and epidermal growth factor receptor (EGFR) mutation status, based on propensity scores derived from IPTW. PD-L1 status and TTF-1 status were not included in the adjustment owing to the high proportion of missing data.

Toxicities were evaluated according to the Common Terminology Criteria for Adverse Events (CTCAE), version 5.0. A two-sided *p*-value of less than 0.05 was considered statistically significant. All statistical analyses were conducted using R software (version 4.3.1; R Foundation for Statistical Computing, Vienna, Austria) and EZR (version 1.68; Saitama Medical Center, Jichi Medical University, Saitama, Japan) [12].

## 3. Results

### 3.1. Patient Characteristics

**Figure 1:**
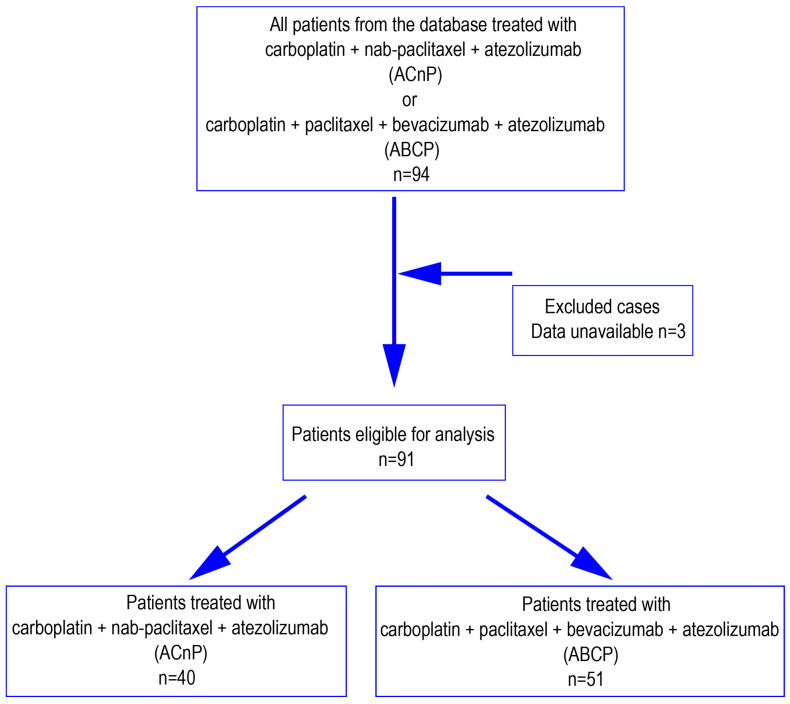
Study flow diagram. ACnP: Atezolizumab combined with carboplatin and nab-. paclitaxel; ABCP: Atezolizumab combined with carboplatin, paclitaxel, and bevacizumab.

The study flow diagram is shown in Figure 1. Based on the predefined treatment regimen, 94 patients were identified from the database. After excluding three cases due to insufficient data, 91 patients were included in the final analysis, comprising 40 in the ACnP group and 51 in the ABCP group. Baseline clinical characteristics of the two groups are presented in Table 1. The ACnP group had a higher proportion of elderly patients compared to the ABCP group. In contrast, the ABCP group had a greater representation of patients with EGFR-mutant lung cancer, females, and never-smokers. The ACnP group comprised a higher proportion of non-adenocarcinoma histological subtypes, including large cell neuroendocrine carcinoma and pleomorphic carcinoma, and exhibited a greater frequency of tumors negative for thyroid transcription factor-1 (TTF-1).

Table 2 summarizes the incidence of dose adjustments across treatment groups. There were no statistically significant differences between the two groups in terms of initial dose reductions or dose reductions during treatment. Notably, 63.4% of patients in the ACnP group skipped at least one scheduled dose of nab-paclitaxel during treatment.

### 3.2. Tumor Response and Disease Control

Objective tumor response and disease control outcomes are summarized in Table 3. The overall response rate (ORR) was 55.0% in the ACnP group and 47.9% in the ABCP group. The disease control rate (DCR) was 85.0% in the ACnP group and 85.4% in the ABCP group. These results indicate that both regimens achieved comparable disease control, despite a numerically higher ORR in the ACnP group.

### 3.3. Survival Outcomes

**Figure 2:**
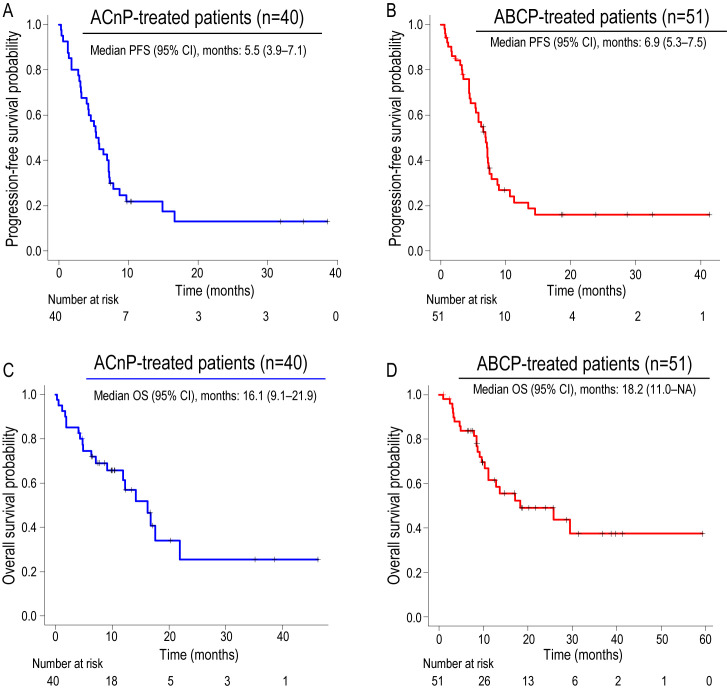
Kaplan–Meier curves of progression-free survival and overall survival in each treatment group. (**A**,**B**) Progression-free survival in the ACnP and ABCP groups. (**C**,**D**) Overall survival in the ACnP and ABCP groups. OS, overall survival; PFS, progression-free survival; ACnP, carboplatin plus nab-paclitaxel plus atezolizumab regimen; ABCP, carboplatin plus paclitaxel plus bevacizumab and atezolizumab regimen.

Kaplan–Meier survival curves for PFS and OS are shown in Figure 2. The median PFS was 5.5 months (95% CI, 4.0–7.1) in the ACnP group and 6.9 months (95% CI, 5.3–7.5) in the ABCP group (Figure 2A,B). Median OS was 16.1 months (95% CI, 9.1–21.9) for the ACnP group and 18.3 months (95% CI, 11.0–not available) for the ABCP group (Figure 2C,D). These findings demonstrate a survival advantage with the ABCP regimen compared with ACnP.

### 3.4. Propensity Score Analysis

**Figure 3:**
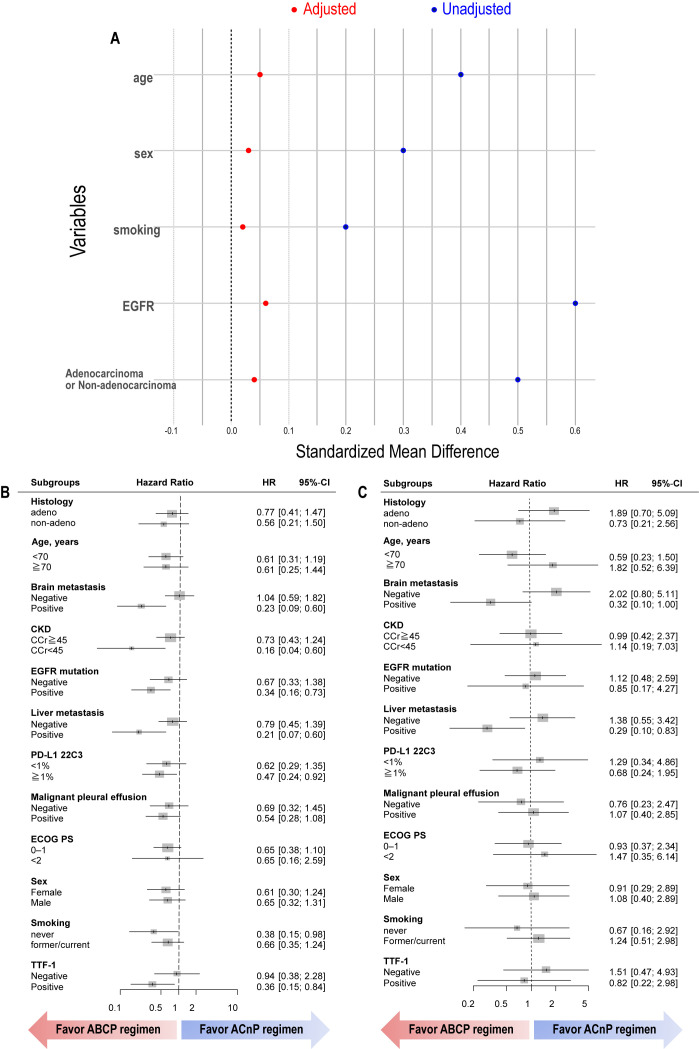
Subgroup analysis of progression-free survival and overall survival adjusted by inverse probability of treatment weighting (IPTW). (**A**), Standardized mean difference (SMD) plots before and after IPTW adjustment. (**B**), Progression-free survival. (**C**), Overall survival. ACnP, carboplatin plus nab-paclitaxel plus atezolizumab regimen; ABCP, carboplatin plus paclitaxel plus bevacizumab and atezolizumab regimen; CKD, chronic kidney disease; ECOG PS, Eastern Cooperative Oncology Group performance status; TTF-1, thyroid transcription factor-1.

Adjustment for potential confounders, including age, sex, smoking history, histology, and EGFR mutation status, was performed using IPTW. Figure 3A presents the standardized mean difference (SMD) plots before and after IPTW adjustment, while Figure 3B,C depicts the subsequent subgroup analyses. For PFS, the ABCP regimen was associated with significantly improved outcomes in patients with brain metastases, liver metastases, EGFR mutations, PD-L1 positivity, and creatinine clearance < 45 mL/min (Figure 3B). For OS, the ABCP regimen demonstrated improved outcomes in patients with brain and liver metastases. These findings highlight the potential clinical value of the ABCP regimen in patient subgroups with traditionally poor prognostic factors.

### 3.5. Safety Profile

Adverse events are summarized in Table 4. The incidence of neutropenia, skin rash, and peripheral neuropathy was lower in the ACnP group. Grade ≥ 3 peripheral neuropathy occurred in 13.7% of patients receiving the ABCP regimen, whereas no cases were observed in the ACnP group, underscoring the higher risk of severe neurotoxicity associated with ABCP. Seven patients discontinued treatment because of immune-related adverse events (irAEs). In the ACnP group, discontinuation was attributed to pneumonitis in two patients and enteritis in one patient. In the ABCP group, one patient developed pneumonitis, one developed a rash, and two experienced liver dysfunction leading to treatment discontinuation.

## 4. Discussion

This retrospective real-world study describes the comparative effectiveness and safety of two distinct first-line treatment regimens, ABCP (atezolizumab, bevacizumab, carboplatin, and paclitaxel) and ACnP (atezolizumab, carboplatin, and nab-paclitaxel), in patients with advanced NSCLC. Our findings indicate that while ABCP demonstrated superior efficacy in aggressive NSCLC subtypes, specifically in patients with liver metastases, brain metastases, and EGFR mutations, this advantage was accompanied by a higher toxicity burden. In contrast, ACnP exhibited a more favorable safety profile, with lower incidences of neutropenia, peripheral neuropathy, and skin rash, highlighting its potential as a well-tolerated alternative, particularly for more vulnerable patient populations.

The observed efficacy of ABCP in EGFR-mutated tumors aligns with a growing body of evidence [8,13,14]. Prior studies, including pivotal trials like IMpower150 [8,13] and ATTLAS, ref. [14] as well as the APPLE trial, ref. [15] have consistently demonstrated the added benefit of bevacizumab to chemotherapy backbones in this molecularly defined subgroup. These findings underscore the potential of anti-angiogenic agents in overcoming the intrinsic resistance to immune checkpoint inhibitors (ICIs) often seen in EGFR-mutated lung cancers [16,17,18]. This resistance is thought to be driven by factors such as low PD-L1 expression, limited tumor-infiltrating lymphocytes, and low tumor mutation burden. Notably, EGFR-mutated tumors often exhibit upregulated VEGF expression, ref. [19] which creates an immunosuppressive microenvironment by hindering dendritic cell maturation, impeding immune cell infiltration, promoting T-cell apoptosis, and fostering the accumulation of immunosuppressive cells like Treg cells and MDSCs [20,21]. The incorporation of bevacizumab in the ABCP regimen likely counteracts this VEGF-mediated immunosuppression, potentially synergizing with the PD-L1 inhibition provided by atezolizumab to enhance antitumor immunity, refs. [22,23] a finding echoed in the improved PFS observed in our EGFR-positive cohort.

Our present study also highlights the potential advantage of ABCP in patients with liver and brain metastases, both recognized as indicators of poor prognosis [24,25]. The involvement of VEGF in the formation of liver metastases, ref. [26] coupled with the observed scarcity of CD8+ T cells in this setting, ref. [27] may explain the potential benefit of bevacizumab-containing regimens. The reported efficacy of atezolizumab and bevacizumab in hepatocellular carcinoma further supports this rationale [28]. Notably, the IMpower150 trial also suggested a trend towards improved OS with ABCP in patients with liver metastases [13]. Similarly, the role of VEGF in the development of brain metastases is well-documented, with bevacizumab demonstrating the ability to inhibit angiogenesis and potentially prevent the growth of micrometastases [29,30,31]. Our findings of improved PFS and OS with bevacizumab in patients with brain metastases further support this evidence.

However, the efficacy gains observed with ABCP must be carefully balanced against its tolerability. Our study revealed a tendency toward a higher incidence of adverse events in the ABCP arm, despite the ACnP group comprising patients with poorer performance status and a higher proportion of elderly individuals. This observation underscores the importance of considering patient-specific factors when selecting a treatment regimen. The ACnP regimen, in contrast, demonstrated a more favorable safety profile, with significantly lower rates of neutropenia, peripheral neuropathy, and skin rash. This improved tolerability aligns with findings from an integrated analysis of IMpower130 and 132, which suggested the benefit of atezolizumab plus chemotherapy even in older patients [32]. Furthermore, the study by Sochinski et al. [33] comparing carboplatin plus paclitaxel versus carboplatin plus nab-paclitaxel in elderly patients suggested that the nab-paclitaxel arm led to better OS due to improved tolerability, allowing for treatment continuation and subsequent lines of therapy. Our findings of reduced hematologic and neurological toxicities with nab-paclitaxel resonate with these observations, suggesting that the ACnP regimen may be a particularly effective and well-tolerated option for elderly patients or those with compromised performance status.

It is important to acknowledge the inherent limitations of our study. The retrospective nature of our analysis introduces the potential for unmeasured confounding factors and limits our ability to establish causality definitively. Furthermore, the relatively small sample size may have limited the statistical power to detect more subtle differences between the treatment groups or within specific subgroups. Of particular concern regarding potential bias is the availability of angiogenesis inhibitors like bevacizumab. As previously suggested, refs. [34,35] patients eligible for and receiving such agents might inherently possess characteristics associated with a better prognosis, irrespective of the treatment itself. This potential selection bias, where clinicians might preferentially prescribe bevacizumab-containing regimens to patients considered more likely to benefit, could have influenced our observed efficacy outcomes. The ACnP regimen evaluated in this study appears to be a suitable alternative for patients who are ineligible for bevacizumab. However, to the best of our knowledge, no previous studies have specifically investigated this regimen in this patient population. Therefore, prospective studies focusing exclusively on bevacizumab-ineligible patients are warranted to confirm these findings. While we employed Inverse Probability of Treatment Weighting (IPTW) to mitigate the impact of observed confounders and reduce this selection bias, it is important to recognize that IPTW is not without its limitations. Despite these limitations, our real-world observations provide valuable hypotheses for future prospective studies. These findings highlight the critical need for personalized treatment strategies in advanced NSCLC, considering both the tumor characteristics and the individual patient profile to optimize outcomes.

## 5. Conclusions

In conclusion, our real-world data provides valuable insights into the comparative landscape of first-line treatment for advanced NSCLC. While the ABCP regimen appears to offer superior efficacy in aggressive subtypes characterized by liver metastases, brain metastases, or EGFR mutations, its higher toxicity burden necessitates careful patient selection. Conversely, the ACnP regimen presents a more favorable safety profile, potentially making it a preferred option for patients who are more vulnerable to treatment-related toxicities, such as elderly individuals or those with poorer performance status. These findings highlight the critical need for personalized treatment strategies in advanced NSCLC, considering both the tumor characteristics and the individual patient profile to optimize outcomes. Future prospective, randomized controlled trials with larger sample sizes are warranted to further validate these real-world observations, address the potential for selection bias, and refine our understanding of the optimal sequencing and selection of these combination regimens.

## Figures and Tables

**Table 1:** Clinical profile of the patients.

	ACnP Group		ABCP Group		*p*-Value
Number of patients	*n* = 40	%	*n* = 51	%	
Median Age [range]	71.5	53–84	67	42–86	0.016
Sex					<0.01
Male	31	77.5	25	49	
Female	9	22.5	26	51	
Smoking					<0.01
Never	6	15	22	43.1	
Former/current	34	85	29	56.9	
ECOG PS					0.15
0	20	50	31	60.8	
1	10	25	15	29.4	
2	8	20	4	7.8	
3	2	5	0	0	
4	0	0	1	2	
Stage					0.651
Recurrence	3	7.5	4	7.8	
4A	10	25	9	17.6	
4B	22	55	35	68.6	
3B	2	5	1	2	
3A	3	7.5	2	3.9	
CKD (CCr < 45)					
Present	6	15	6	11.8	0.759
None	34	85	45	88.2	
Histology					0.031
Adenocarcinoma	24	60	40	81.6	
Not adenocarcinoma	16	40	8	18.4	
Malignant pleural effusion					0.397
Positive	16	40	26	51	
Negative	24	60	25	49	
Liver metastasis					1
Positive	8	20	11	21.6	
Negative	32	80	40	78.4	
Brain metastasis					0.822
Positive	12	30	17	33.3	
Negative	28	70	34	66.7	
EGFR mutations					<0.01
Positive	5	13.2	33	64.7	
Negative	33	86.8	18	35.3	
PD-L1 TPS					0.442
Negative	10	25	18	35.3	
Low (1–49%)	15	37.5	21	41.2	
High (≧50%)	10	25	8	15.7	
Unknown	5	12.5	4	7.8	
TTF-1					0.021
Positive	11	27.5	21	41.2	
Negative	19	47.5	10	19.6	
Unknown	10	25	20	39.2	
Discontinuation due to adverse events	3	7.5	4	7.8	

ECOG: Eastern Cooperative Oncology Group, PS: performance status. TTF-1: Thyroid transcription factor-1. ACnP: carboplatin plus nab-paclitaxel plus atezolizumab regimen. ABCP: carboplatin plus paclitaxel plus bevacizumab and atezolizumab regimen.

**Table 2:** Frequency of dose reduction and nab-paclitaxel skipping.

	ACnP Group (%)	ABCP Group
Number of patients	40	51
Dose reduction status		
With reduction	34 (85%)	36 (70.6%)
Without reduction	6 (15%)	15 (29.4%)
nabPTX dose skipping	26 (63.4%)	

Data are presented as counts (percentages). ACnP: carboplatin plus nab-paclitaxel plus atezolizumab regimen, ABCP: carboplatin plus paclitaxel plus bevacizumab and atezolizumab regimen. Nab-paclitaxel (nabPTX) was not included in the ABCP regimen, so skipping data is only applicable to the ACnP group.

**Table 3:** Tumor response rate and disease control rate.

	ACnP Group	ABCP Group
Number of patients	40	51
Complete response	1 (2.5%)	2 (3.9%)
Partial response	21 (52.5%)	21 (41.2%)
Stable disease	12 (30%)	18 (35.3%)
Progressive disease	6 (15%)	7 (13.7%)
Not evaluated	0 (0%)	3 (5.9%)
Overall response rate	22 (55%)	23 (47.9%)
Disease control rate	34 (85%)	41 (85.4%)

Data are presented as counts (percentages). ACnP: carboplatin plus nab-paclitaxel plus atezolizumab regimen, ABCP: carboplatin plus paclitaxel plus bevacizumab and atezolizumab regimen.

**Table 4:** Adverse events.

	ACnP Group (*n* = 40)		ABCP Group (*n* = 51)	
	Any Grade (%)	Grade ≥ 3	Any Grade (%)	Grade ≥ 3
Leukopenia	25 (62.5%)	9 (22.5%)	44 (86.3%)	35 (68.6%)
Neutropenia	24 (60%)	11 (27.5%)	46 (90.2%)	39 (76.5%)
Anemia	36 (90%)	7 (17.5%)	42 (82.4%)	8 (15.7%)
Thrombocytopenia	19 (47.5%)	4 (10%)	34 (66.7%)	6 (11.8%)
Liver dysfunction	10 (25%)	1 (2.5%)	15 (29.4%)	5 (9.8%)
Enterocolitis	3 (7.5%)	0	1 (2%)	0
Pneumonitis	4 (10%)	1 (2.5%)	6 (11.8%)	2 (3.9%)
Hypothyroidism	4 (10%)	0	1 (2%)	0
Adrenal insufficiency	0	0	0	0
Glucose intolerance	1 (2.5%)	0	2 (3.9%)	1 (2%)
Skin rash	1 (2.5%)	0	14 (27.5%)	5 (9.8%)
Myositis/Myocarditis	0	0	1 (2%)	0
Peripheral neuropathy	6 (15%)	0	28 (54.9%)	7 (13.7%)
Epistaxis	0	0	3 (5.9%)	1 (2%)
Pulmonary embolism	0	0	1 (2%)	1 (2%)
Gastrointestinal hemorrhage	0	0	1 (2%)	1 (2%)

Data are presented as counts (percentages). ACnP: carboplatin plus nab-paclitaxel plus atezolizumab regimen, ABCP: carboplatin plus paclitaxel plus bevacizumab and atezolizumab regimen.

## Data Availability

All data generated or analyzed during the current study are included in this article. Additional data and materials are available from the first author (Ayaka Ohiwa) or the second author (Tadashi Nishimura) upon reasonable request.
